# Post-Graduate Examinations

**Published:** 1911-09-16

**Authors:** 


### Post-Graduate Examinations.

When the medical student after years of toil at last circumvents his final examination and becomes a qualified medical man, his gratification at becoming a registered medical practitioner is hardly greater than his thankfulness at being rid of the examination incubus. The relief with which he turns his back on examination halls once and for all is a feeling the strength of which perhaps only those who have once been medical students can fully appreciate; for surely there is no other profession so beset with examinations as is medicine. There are, as is well known, plenty of clear-headed people who rail at the examination system, and would see it abolished or at least very much cut down; nevertheless, the system continues to flourish and even to extend itself; and in medicine it seems to be as firmly planted as anywhere else.

If American doctors are anything like ourselves, they must have had a shock when they read the address of the President of the American Medical Association, which appears in the Journal of the Association for July 1st. Dr. Murphy advances several suggestions for the improvement of the profession in its relations with the problems of the life and health of the ordinary citizen, and some of them are both ingenious and admirable. The passages to which we at present refer are those in which he proposes that medical men should be required, at the expiration of five or ten years from the date of qualification, to pass another examination; and in case of failure to take another prescribed course of study until they do pass! Revolutionary as this idea appears, and impracticable as it would no doubt prove *at present*, it is not merely a *jeu d'esprit* that can be entirely neglected; for there is a good deal to be said in favour of it, as a short reflection will soon show.

The mere existence of such a regulation would keep up to the mark that not inconsiderable section of the profession which without shame never reads a text-book or a medical paper, once it has obtained a licence to practise. It would raise the general standard of efficiency right through the profession. The conscientious, hard-working clinician would have nothing to fear; the black sheep, on the contrary, would either be forced to leave the profession or else to improve his knowledge of it. It is almost idle to elaborate the details of a plan for which (though we believe it to be admirable in principle) the times are not yet ripe. But it is easy to see that it might prove the solution of the many-portal difficulty in medicine. If the examining corporations as they at present exist were not allowed to carry out this post-graduate examination, but the duty were made to devolve on the General Medical Council, many of the advantages of the one-portal system would be secured without much disturbance of the present teaching and licensing corporate bodies. Further, the level of efficiency of the latter would be speedily raised; for students would not flock to the University of X, easy though the M.B. (X) may be of attainment, if it became obvious that graduates of X failed at their post-graduate examination in unduly large numbers. The more this suggestion of Dr. Murphy's is pondered, the more fascinating it seems; though we do not expect that the prospect of facing an examiner once more raises joyful expectancy in the heart of any of us individually. Amongst the general public it would not be at all astonishing to hear of the scheme being hailed with acclamation.

